# Association between serum lactate level during cardiopulmonary resuscitation and survival in adult out-of-hospital cardiac arrest: a multicenter cohort study

**DOI:** 10.1038/s41598-020-80774-4

**Published:** 2021-01-15

**Authors:** Norihiro Nishioka, Daisuke Kobayashi, Junichi Izawa, Taro Irisawa, Tomoki Yamada, Kazuhisa Yoshiya, Changhwi Park, Tetsuro Nishimura, Takuya Ishibe, Yoshiki Yagi, Takeyuki Kiguchi, Masafumi Kishimoto, Toshiya Inoue, Yasuyuki Hayashi, Taku Sogabe, Takaya Morooka, Haruko Sakamoto, Keitaro Suzuki, Fumiko Nakamura, Tasuku Matsuyama, Yohei Okada, Satoshi Matsui, Atsushi Hirayama, Satoshi Yoshimura, Shunsuke Kimata, Takeshi Shimazu, Tetsuhisa Kitamura, Takashi Kawamura, Taku Iwami, the CRITICAL Study Group Investigators, Norihiro Nishioka, Daisuke Kobayashi, Junichi Izawa, Taro Irisawa, Tomoki Yamada, Kazuhisa Yoshiya, Changhwi Park, Tetsuro Nishimura, Takuya Ishibe, Yoshiki Yagi, Takeyuki Kiguchi, Masafumi Kishimoto, Toshiya Inoue, Yasuyuki Hayashi, Taku Sogabe, Takaya Morooka, Haruko Sakamoto, Keitaro Suzuki, Fumiko Nakamura, Tasuku Matsuyama, Yohei Okada, Satoshi Matsui, Atsushi Hirayama, Satoshi Yoshimura, Shunsuke Kimata, Takeshi Shimazu, Tetsuhisa Kitamura, Takashi Kawamura, Taku Iwami

**Affiliations:** 1 Department of Preventive Services, Kyoto University School of Public Health, Kyoto, Japan; 2 Kyoto University Health Service, Yoshida-Honmachi, Sakyo-ku, Kyoto, 606-8501 Japan; 3 Department of Medicine, Okinawa Prefectural Yaeyama Hospital, Ishigaki, Japan; 4 Department of Traumatology and Acute Critical Medicine, Osaka University Graduate School of Medicine, Suita, Japan; 5 Emergency and Critical Care Medical Center, Osaka Police Hospital, Osaka, Japan; 6 Department of Emergency and Critical Care Medicine, Kansai Medical University, Takii Hospital, Moriguchi, Japan; 7 Department of Emergency Medicine, Tane General Hospital, Osaka, Japan; 8 Department of Critical Care Medicine, Osaka City University, Osaka, Japan; 9 Department of Emergency and Critical Care Medicine, Kindai University Faculty of Medicine, Osaka-Sayama, Japan; 10 Osaka Mishima Emergency Critical Care Center, Takatsuki, Japan; 11 Critical Care and Trauma Center, Osaka General Medical Center, Osaka, Japan; 12 Osaka Prefectural Nakakawachi Medical Center of Acute Medicine, Higashi-Osaka, Japan; 13 Senshu Trauma and Critical Care Center, Osaka, Japan; 14 Senri Critical Care Medical Center, Saiseikai Senri Hospital, Suita, Japan; 15 Traumatology and Critical Care Medical Center, National Hospital Organization Osaka National Hospital, Osaka, Japan; 16 Emergency and Critical Care Medical Center, Osaka City General Hospital, Osaka, Japan; 17 Department of Pediatrics, Osaka Red Cross Hospital, Osaka, Japan; 18 Emergency and Critical Care Medical Center, Kishiwada Tokushukai Hospital, Osaka, Japan; 19 Department of Emergency and Critical Care Medicine, Kansai Medical University, Hirakata, Osaka, Japan; 20 Department of Emergency Medicine, Kyoto Prefectural University of Medicine, Kyoto, Japan; 21 Division of Environmental Medicine and Population Sciences, Department of Social and Environmental Medicine, Graduate School of Medicine, Osaka University, Osaka, Japan; 22 Public Health, Department of Social and Environmental Medicine, Osaka University Graduate School of Medicine, Osaka, Japan

**Keywords:** Cardiovascular diseases, Cardiology, Prognostic markers

## Abstract

We aimed to investigate the association between serum lactate levels during cardiopulmonary resuscitation (CPR) and survival in patients with out-of-hospital cardiac arrest (OHCA). From the database of a multicenter registry on OHCA patients, we included adult nontraumatic OHCA patients transported to the hospital with ongoing CPR. Based on the serum lactate levels during CPR, the patients were divided into four quartiles: Q1 (≤ 10.6 mEq/L), Q2 (10.6–14.1 mEq/L), Q3 (14.1–18.0 mEq/L), and Q4 (> 18.0 mEq/L). The primary outcome was 1-month survival. Among 5226 eligible patients, the Q1 group had the highest 1-month survival (5.6% [74/1311]), followed by Q2 (3.6% [47/1316]), Q3 (1.7% [22/1292]), and Q4 (1.0% [13/1307]) groups. In the multivariable logistic regression analysis, the adjusted odds ratio of Q4 compared with Q1 for 1-month survival was 0.24 (95% CI 0.13–0.46). 1-month survival decreased in a stepwise manner as the quartiles increased (*p* for trend < 0.001). In subgroup analysis, there was an interaction between initial rhythm and survival (*p* for interaction < 0.001); 1-month survival of patients with a non-shockable rhythm decreased when the lactate levels increased (*p* for trend < 0.001), but not in patients with a shockable rhythm (*p* for trend = 0.72). In conclusion, high serum lactate level during CPR was associated with poor 1-month survival in OHCA patients, especially in patients with non-shockable rhythm.

## Introduction

Out-of-hospital cardiac arrest (OHCA) represents one of the serious public health concerns in industrialized countries, with approximately 350,000–700,000 deaths due to OHCA annually in the European Union^1^, 400,000 in the United States^2^, and 120,000 in Japan^3^. Recently, advanced interventions before the return of spontaneous circulation (ROSC), such as the implementation of extracorporeal membrane oxygenation (ECMO) and target temperature management (TTM), have been attempted to improve the prognosis of cardiac arrest patients^4,5^. Nonetheless, it is not easy for emergency and/or intensive care physicians to decide whether to proceed to the advanced interventions during cardiopulmonary resuscitation (CPR) or to stop CPR during resuscitation efforts for OHCA patients who do not achieve ROSC^6,7^. Therefore, a marker, which can be measured during CPR, can facilitate the decision-making process in the provision of intensive care for OHCA patients to improve their outcomes.

Lactate levels can be measured easily and quickly in clinical settings and represent a good marker of tissue hypoxia^8–10^. Therefore, lactate levels can be measured to predict prognosis in critical illnesses such as sepsis, trauma, and burns^11–13^. On the other hand, the relation between serum lactate levels in the early phase after ROSC and outcome of cardiac arrest patients has been controversial^15–19^. Additionally, the usefulness of lactate levels during CPR without achieving ROSC in OHCA patients, as a predictor, has not been sufficiently evaluated.

Using a multicenter, prospective registry in Osaka, Japan, designed to accumulate both pre- and in-hospital information on treatments and laboratory data of OHCA patients, this study aimed to investigate the association between serum lactate levels during CPR and survival outcomes in OHCA patients who did not achieve ROSC in prehospital settings.

## Methods

### Study design and setting

The present study was a retrospective analysis of data from the Comprehensive Registry of Intensive Cares for OHCA Survival (the CRITICAL) study. The CRITICAL study is a multicenter prospective registry, designed to accumulate both the prehospital and the in-hospital data on OHCA treatments, to understand the entire care process, and to improve outcomes after OHCA. A complete description of the study methodology can be found elsewhere^20^. The CRITICAL study group consists of 16 tertiary emergency medical institutions including 15 Critical Care Medicine Centers (CCMCs) and 1 non-CCMC with a department of emergency treatments in Osaka Prefecture in Japan, which has an area of 1905 km^2^ and had a residential population of about 8.8 million in 2017. Approximately 7500 OHCA cases occur in Osaka Prefecture every year^21^ and nearly 30% of them are transported to the CCMCs^22^. Therefore, over 2000 OHCA patients have been registered every year, and the study is ongoing with an indefinite study period. The CRITICAL study registered consecutive patients suffering from OHCA, who were resuscitated by emergency medical services (EMS) and were transported to the participating hospitals. This registry excluded OHCA patients for whom CPR was not performed by the physicians after arrival at the hospital or whose family members refused to participate in the registry.

### Study population

In this study, we included adult patients aged ≥ 18 years with OHCA with non-traumatic causes, who did not achieve ROSC before arrival at the hospital, and whose serum lactate levels during CPR at the hospital were measured during the study period from January 1, 2013 to December 31, 2017. We excluded OHCA patients whose ECMO was initiated before the lactate measurement. This study protocol was approved by the Ethics Committee of Kyoto University and each participating hospital.

### The emergency medical service system in Osaka Prefecture

Anyone can call the 119 emergency services anywhere in Japan, and on receipt of the emergency call, a dispatch center sends the nearest available ambulance to the scene. Emergency services are accessible anytime. Each ambulance includes a three-person unit providing life support. They can insert an intravenous line and an adjunct airway and use a semi-automated external defibrillator for OHCA patients. Highly trained EMS personnel are called emergency life-saving technicians, who are allowed to provide shocks without consulting a physician, and specially trained emergency life-saving technicians are permitted to perform tracheal intubation to administer adrenaline to OHCA patients. Since EMS providers cannot generally accept do-not-resuscitate orders in Japan, they do not terminate resuscitation in the field. Therefore, the EMS personnel transports almost all OHCA patients, except cases of decapitation, incineration, decomposition, rigor mortis, or dependent cyanosis. All EMS providers perform CPR according to the Japanese CPR guidelines^23^. Previous reports have illustrated details of the EMS system in Japan^24,25^.

### Prehospital and in-hospital measurements

We obtained anonymized prehospital resuscitation data from the All-Japan Utstein Registry of the Fire and Disaster Management Agency (FDMA). Details of the registry have been described in previous papers^2,26^. Data were collected prospectively using the data form of the Utstein-style international guideline of reporting OHCA^27^. The following data were collected: witness status, bystander-initiated CPR, shock delivery by public-access automated external defibrillators, dispatcher instructions, first documented rhythm on the EMS arrival, advanced airway management, adrenalin administration, and resuscitation time courses. A data form was completed by the physician in charge of the patient or medical staff in cooperation with the physician. Data were uploaded into the registry system on the FDMA database server, checked logically by the computer system, and confirmed by the implementation working group. If a data form was incomplete, the FDMA returned it to the specific fire station for completion.

In this registry, detailed data were prospectively collected for consecutive patients with OHCA after they arrived at the hospital. Anonymized data were entered into a web form by the physician or medical staff in cooperation with the physician in charge of the patient, checked logically by the computer system, and confirmed by the CRITICAL study working group. If a data form was incomplete, the working group returned it to the specific institution for completion. The working group combined the detailed in-hospital data and the Utstein-style data from the FDMA on the web, based on five key variables of the emergency call: date, time, age, sex, and Glasgow-Pittsburgh cerebral performance category (CPC) present in both datasets.

In-hospital data of OHCA patients after arrival at the hospital were collected prospectively using the original report form: age, ROSC after hospital arrival, first documented rhythm at the hospital, laboratory data, actual detailed treatments for an OHCA patient (e.g., ECMO, coronary angiography (CAG), and TTM), causes of arrest, and outcome data. Blood gases were measured upon arrival at the hospital among eligible patients. The causes of arrest were divided into cardiac and non-cardiac (cerebrovascular diseases, respiratory diseases, malignant tumors, and external causes, including traffic injury, fall, hanging, drowning, asphyxia, drug overdose, or any other external cause) origins. The diagnosis of cardiac or non-cardiac origin was made clinically by the physician in charge. Regarding the outcome data, 1-month survival and neurological status were prospectively collected.

### Outcomes

The primary outcome of this study was 1-month survival. The secondary outcomes were any ROSC after hospital arrival and 30-day survival with a good neurological outcome, defined as CPC of 1 or 2. We defined ROSC as a continuous palpable circulation with a self-beat for more than 30 s^27^. The neurological status was evaluated by the physician responsible for treating the patient using the CPC scale (category 1, good cerebral performance; category 2, moderate cerebral disability; category 3, severe cerebral disability; category 4, coma or vegetative state; category 5, death/brain death)^28^.

### Statistical analysis

In this study, we divided patients into quartiles based on the serum lactate levels with on-going CPR: Q1 (lactate ≤ 10.6 mmol/L), Q2 (10.6 < lactate ≤ 14.1 mmol/L), Q3 (14.1 < lactate ≤ 18.0 mmol/L), and Q4 (lactate > 18.0 mmol/L). We evaluated the patient characteristics between the four groups using Kruskal–Wallis tests for continuous variables and chi-square tests or Fisher exact tests for categorical variables. In addition, to determine the association between the lactate level quartiles and outcomes, we constructed univariable and multivariable logistic regression models and assessed crude and adjusted odds ratios (OR) and their 95% confidence intervals (CIs). The Cochran-Armitage trend test was used for the trend analysis across the lactate level quartiles. We adjusted for the potential confounders in the multivariable models that were biologically and clinically essential and considered to be associated with the outcomes, including age (continuous value), sex (men or women), witness status (yes or no), bystander CPR status (yes, no), first documented rhythm at the scene (shockable [ventricular fibrillation and pulseless ventricular tachycardia] or non-shockable [pulseless electrical activity and asystole]), prehospital adrenaline administration (yes or no), prehospital advanced airway management (yes or no), and time from the call to lactate measurement (continuous value). We did a complete case analysis with adjusted confounders. In a sensitivity analysis, we also conducted the multivariable logistic analysis adjusted for advanced procedures such as ECMO (yes or no), TTM (yes or no), and CAG (yes or no). Additionally, we performed a receiver operating characteristics (ROC) curve analysis with an area under the curve of serum lactate levels before ROSC to predict 1-month survival in patients with OHCA. Moreover, we carried out a planned subgroup analysis stratified by the first documented rhythm at the scene (shockable or non-shockable). We evaluated the patient characteristics between the shockable and non-shockable rhythm groups using Kruskal–Wallis tests for continuous variables and chi-square tests or Fisher exact tests for categorical variables. We assessed the interaction effect between lactate levels and first documented rhythm in 1-month survival in the multivariable logistic regression model. All *p* values were two-tailed and 0.05 levels were considered statistically significant. All statistical analyses were conducted using R (The R Foundation for Statistical Computing, version 3.6.0) and EZR (Saitama Medical Center, Jichi Medical University, version 1.41, Saitama, Japan), which is a graphical user interface for R^29^.

### Ethics approval and consent to participate

The Ethics Committee of Kyoto University Graduate School of Medicine and participating institution approved this registry study (R1045). The written informed consent requirement was waived by Ethics Committee of Kyoto University Graduate School because only de-identified data were used.

## Results

### Study participants

**Figure 1:**
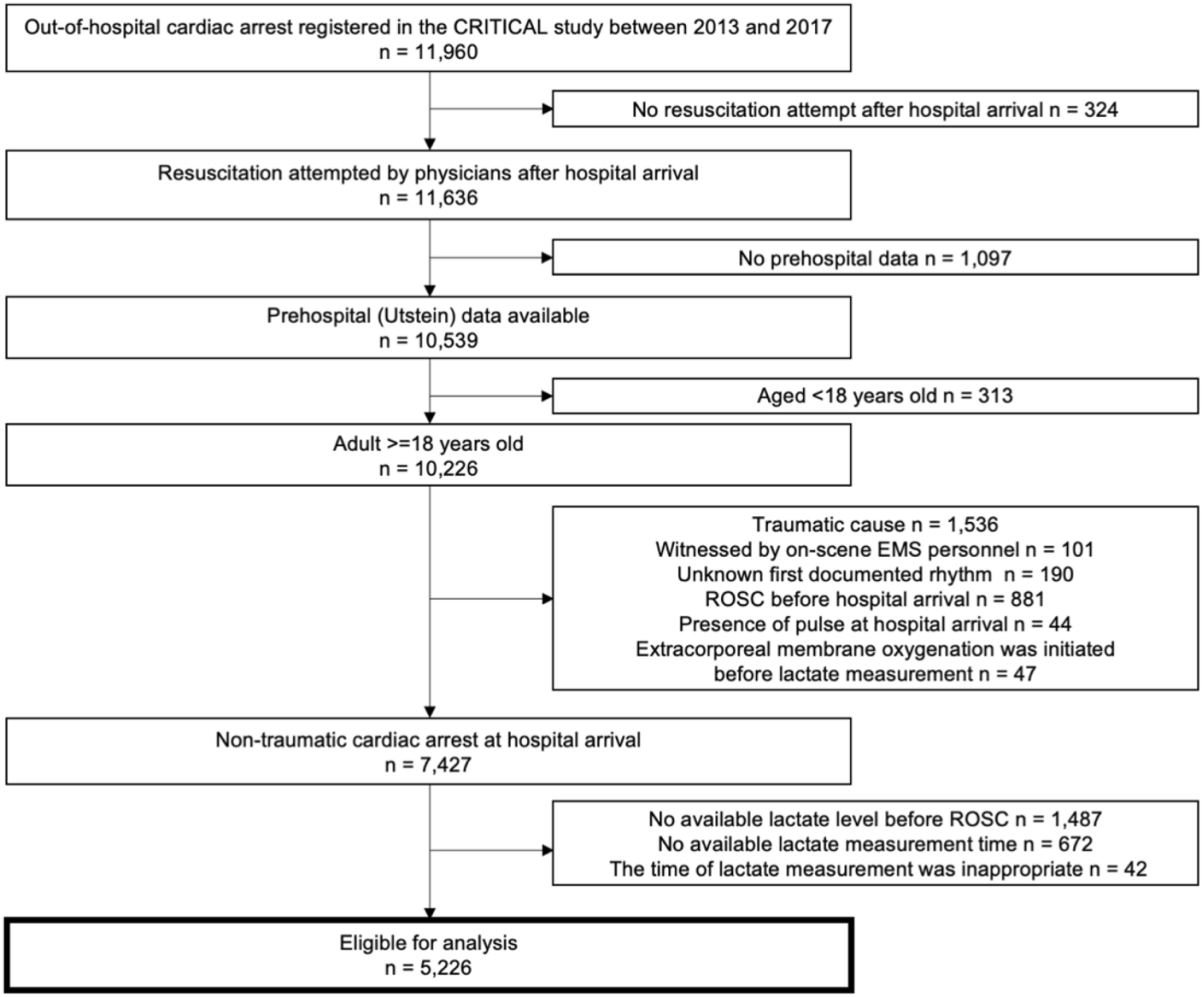
Overview of out-of-hospital cardiac arrests from 2013 to 2017 in the CRITICAL study. *EMS* emergency medical service, *OHCA* out-of-hospital cardiac arrest, *ROSC* return of spontaneous circulation.

A total of 11,960 OHCA patients were documented during the study period. Of the 11,636 patients in whom resuscitation was attempted after arrival at the hospital, the data of 10,539 patients were combined with the prehospital data. Of them, we excluded 313 children aged < 18 years, 1536 patients with traumatic causes, 101 patients whose OHCA was witnessed by on-scene EMS personnel, 190 patients whose first documented rhythm was unknown, 881 patients who achieved ROSC in prehospital settings, 44 patients who presented the pulse at hospital arrival, and 47 patients for whom ECMO was initiated before lactate measurement. Further excluding 1487 patients without data on serum lactate data before ROSC, 672 patients without data on time to lactate measurement, and 47 patients with the inappropriate time interval from measuring lactate to ROSC, a total of 5226 OHCA patients who were admitted to the hospital without ROSC were finally eligible for our analyses (Fig. 1).

The characteristics of patients divided by lactate level quartile are outlined in Table 1. In the Q1 group, the median age was higher than that in other groups. The Q1 group was more likely to have witnessed status, adrenaline administration by EMS, first documented shockable rhythm at the scene, shockable rhythm on hospital arrival, CAG, and TTM than other groups. Furthermore, the time from the call to arrival at the hospital and the time from call to lactate measurement were shorter in the Q1 than in the other quartiles, but the time from arrival at the hospital to measuring lactate was similar for all the groups.

**Table 1:** Characteristics after OHCA patients by serum lactate level measured during cardiac arrest at hospital arrival.

	All Patients (n = 5226)	Missing	Quartile 1 (≤ 10.6 mmol/L) (n = 1311)	Quartile 2 (10.6–14.1 mmol/L) (n = 1316)	Quartile 3 (14.1–18 mmol/L) (n = 1292)	Quartile 4 (> 18 mmol/L) (n = 1307)	*p* values*
**Patient characteristics**							
Age, median [IQR]	75 (64–83)	0 (0.0)	76 (67–83)	76 (65–83)	75 (64–83)	71 (56–80)	< 0.001
Sex, male, n (%)	3246 (62.1)	0 (0.0)	798 (60.9)	826 (62.8)	811 (62.8)	811 (62.1)	0.72
Witnessed arrest, n (%)	2324 (44.5)	0 (0.0)	815 (62.2)	666 (50.6)	501 (38.8)	276 (23.8)	< 0.001
Cardiac etiology of arrest, n (%)	3581 (68.5)	0 (0.0)	861 (65.7)	890 (67.6)	905 (70.0)	925 (70.8)	< 0.001
**Pre-hospital information**							
Bystander CPR, n (%)	2198 (42.1)	0 (0.0)	518 (39.5)	564 (42.9)	517 (40.0)	599 (45.8)	0.003
Shock by public-access AEDs, n (%)	53 (1.0)	0 (0.0)	7 (0.5)	22 (1.7)	18 (1.4)	6 (0.5)	0.002
Shockable rhythm at the scene, n (%)	509 (9.7)	0 (0.0)	181 (13.8)	158 (12.0)	99 (7.7)	71 (5.4)	< 0.001
Adrenaline administration by EMS, n (%)	910 (17.4)	0 (0.0)	274 (20.9)	243 (18.5)	239 (18.5)	154 (11.8)	< 0.001
Advanced airway management, n (%)	2268 (43.4)	0 (0.0)	560 (42.7)	575 (43.7)	585 (45.3)	548 (41.9)	0.347
Time from call to EMS contact, median (IQR), min	8 (7–10)	7 (0.1)	8 (6–10)	8 (6–10)	8 (7–10)	8 (7–10)	< 0.001
Time from call to hospital arrival, median (IQR), min	32 (27–40)	56 (1.1)	31 (26–39)	32 (26–39)	33 (27–41)	33 (28–42)	< 0.001
**In-hospital information**							
Shockable rhythm on hospital arrival, n (%)	272 (5.2)	0 (0.0)	86 (6.6)	96 (7.3)	49 (3.8)	41 (3.1)	< 0.001
Adrenaline administration at hospital, n (%)	4827 (92.4)	11 (0.2)	1207 (92.3)	1214 (92.5)	1204 (93.4)	1202 (92.0)	0.553
Extracorporeal membrane oxygenation, n (%)	248 (4.8)	0 (0.0)	67 (5.1)	75 (5.7)	66 (5.1)	40 (3.1)	0.009
Coronary angiography, n (%)	258 (4.9)	0 (0.0)	74 (5.6)	84 (6.4)	70 (5.4)	30 (2.3)	< 0.001
Target temperature management, n (%)	211 (4.0)	0 (0.0)	72 (5.5)	70 (5.3)	41 (3.2)	28 (2.1)	< 0.001
Time from call to lactate measurement, median (IQR), min	40 (32–51)	56 (1.1)	38 (30–49)	39 (32–51)	41 (34–53)	42 (34–54)	< 0.001
Time from hospital arrival to lactate measurement, median (IQR), min	5 (3–10)	0 (0.0)	5 (2–9)	5 (2–10)	6 (3–11)	6 (3–12)	< 0.001

*OHCA* out-of-hospital cardiac arrest, *ROSC* return of spontaneous circulation, *AED* automated external defibrillator, *CPR* cardiopulmonary resuscitation, *EMS* emergency medical services, *IQR* Inter quartile range.

*Comparisons between the four groups were evaluated with Kruskal–Wallis tests for continuous variables and χ^2^ test for categorical variables.

The characteristics of patients by the initial documented rhythm are shown in Supplementary Table 1. The shockable rhythm group was more likely younger, male, to have witnessed arrest, to have cardiac cause, administered with adrenaline by EMS, to undergo ECMO, CAG, and TTM. The time from call to lactate measurement and the time from arrival at the hospital to measuring lactate were similar for both groups. Lactate level before ROSC in the non-shockable rhythm group was higher than the shockable rhythm group.

### Outcomes

Overall, 1-month survival was 2.9% (156/5226). The rates of 1-month survival decreased gradually from the first to the fourth quartile (Q1, 5.6%; Q2, 3.6%; Q3, 1.7%; Q4, 1.0% (*p* for trend < 0.001; Table 2). In the multivariable logistic regression analysis, 1-month survival in the highest quartile group was significantly lower than that in the lowest quartile group (adjusted OR 0.24; 95% CI 0.13–0.46). The ROC curve showed the area under the curve of 0.68 (95% CI 0.64–0.72). The proportion of favorable neurological outcomes showed a similar tendency as 1-month survival (p for trend = 0.0014). However, we could not investigate the neurological outcomes by multivariable analysis because of small number of survivors with a favorable neurological outcome after hospital arrival.

**Table 2:** Outcomes after OHCA by serum lactate level measured during cardiac arrest at hospital arrival.

	Quartile 1 (≤ 10.6 mmol/L)	Quartile 2 (10.6–14.1 mmol/L)	Quartile 3 (14.1–18 mmol/L)	Quartile 4 (> 18 mmol/L)	*p* for trend
	n = 1311	n = 1316	n = 1292	n = 1307	
**Primary outcome**					
One-month survival (%)	74 (5.6)	47 (3.6)	22 (1.7)	13 (1.0)	< 0.001
Crude OR (95% CI)	Reference	0.62 (0.43–0.90)	0.29 (0.18–0.47)	0.17 (0.093–0.30)	
Adjusted OR (95% CI)*	Reference	0.53 (0.27–0.92)	0.36 (0.22–0.60)	0.24 (0.13–0.46)	
**Secondary outcome**					
Any ROSC after measuring lactate (%**)**	457 (34.9)	430 (32.7)	323 (25.0)	193 (14.8)	< 0.001
Crude OR (95% CI)	Reference	0.91 (0.77–1.07)	0.62 (0.53–0.74)	0.32 (0.27–0.39)	
Adjusted OR (95% CI)*	Reference	1.04 (0.88–1.23)	0.81 (0.68–0.97)	0.46 (0.37–0.56)	
Favorable neurological outcome (%)	24 (1.8)	17 (1.3)	10 (0.8)	8 (0.6)	0.0014
Crude OR (95% CI)	Reference	0.70 (0.38–1.31)	0.42 (0.20–0.88)	0.33 (0.15–0.74)	
Adjusted OR	–	–	–	–	–

*OHCA* out-of-hospital cardiac arrest, *ROSC* return of spontaneous circulation, *OR* odds ratio, *CI* confidence interval.

*Adjusted for age, sex, bystander witness, bystander CPR, first documented rhythm at the scene, prehospital advanced airway management, prehospital adrenaline administration, and time from EMS call to lactate measurement.

In a sensitivity analysis adjusted for advanced procedures, we observed similar results, and the proportion of l-month survival outcome in the highest quartile group was lower compared with that in the lowest quartile group (adjusted OR 0.22; 95% CI 0.11–0.46; Supplementary Table 2).

In the subgroup analysis stratified by the first documented rhythm (Table 3), 1-month survival of OHCA patients with non-shockable rhythm decreased when the lactate levels increased (*p* for trend < 0.001), but in patients with shockable rhythm, this trend was not observed (*p* for trend = 0.72). There was a significant interaction (*p* for interaction < 0.001) between the rhythms and lactate levels in predicting 1-month survival. The ROC curve showed area under the curve of 0.75 (95% CI 0.70–0.80) in patients with non-shockable rhythm and of 0.51 (95% CI 0.44–0.58) in patients with shockable rhythm.

**Table 3:** One-month survival after OHCA by serum lactate level measured during cardiac arrest at hospital arrival according to first documented rhythm.

	Quartile 1 (≤ 10.6 mmol/L)	Quartile 2 (10.6–14.1 mmol/L)	Quartile 3 (14.1–18 mmol/L)	Quartile 4 (> 18 mmol/L)	*p* for trend	*p* for interaction*
**Shockable rhythm (n** **=** **509)**						< 0.001
n/N (%)	28/181 (15.5)	24/158 (15.2)	16/99 (16.2)	9/71 (12.7)	0.72	
Crude OR (95% CI)	Reference	0.98 (0.54 to 1.77)	1.05 (0.54 to 2.06)	0.79 (0.35 to 1.78)		
**Non shockable rhythm (n** **=** **4717)**						
n/N (%)	46/1130 (4.1)	23/1158 (2.0)	6/1193 (0.5)	4/1236 (0.3)	< 0.001	
Crude OR (95%CI)	Reference	0.48 (0.29 to 0.79)	0.12 (0.051 to 0.28)	0.077 (0.028 to 0.21)		

*OHCA* out-of-hospital cardiac arrest, *ROSC* return of spontaneous circulation, *OR* odds ratio, *CI* confidence interval.

*p for interaction was calculated between serum lactate level and first documented rhthm in 1-month survival.

## Discussion

### Summary

Using a Japanese large-scale prospective registry of OHCA, we demonstrated that the increased serum lactate level during CPR was significantly associated with decreased 1-month survival among OHCA patients. Especially, the relationship between high lactate levels during CPR and decreased 1-month survival was observed in patients with non-shockable initial rhythm, but not in patients with shockable rhythm. Our results elucidating the association between lactate levels before ROSC and outcomes after cardiac arrest suggest that lactate levels may serve as one of the prognostic biomarkers of OHCA.

### Comparison with previous studies

Previous studies on serum lactate levels focused mainly on OHCA patients who achieved ROSC. Lee and colleagues, in South Korea, reported that high lactate levels within 1 h after ROSC were related to hospital mortality and poor neurological outcomes in OHCA patients who underwent therapeutic hypothermia^15^. Moreover, Shinozaki and colleagues, in Japan, revealed that lactate and blood ammonia levels within 15 min of hospital arrival were independent prognostic factors, and when both biomarker levels were over the threshold, the positive predictive value for poor outcomes was nearly 100%^16^. There were few studies regarding serum lactate levels before ROSC. Sarıaydın and colleagues, in Turkey, found that initial lactate levels of OHCA patients without ROSC before hospital arrival were not associated with 24-h survival, but the number of patients was small^30^. Wang and colleagues in Taiwan reported that high pre-ROSC lactate level has also been shown to correlate with poor outcomes in patients with in-hospital cardiac arrest^31^. On the other hand, as for OHCA, most of the previous studies evaluated not to distinguish lactate levels between OHCA patients before and after ROSC, and this is the first to assess the association between lactate during CPR and the outcomes in OHCA. Importantly, our large-scale registry enabled us to demonstrate the dose-dependent association between serum lactate levels during CPR and 1-month survival among OHCA patients by multivariable logistic regression analysis.

### Interpretation of the results and possible implications

Lactate levels are considered to be predictive markers of hemodynamic indicators in critical illness^8–10^. Cardiac arrest causes systemic tissue hypoxia, consequently resulting in the production of excessive lactate levels in anaerobic metabolism^8–10^. Even with chest compressions during cardiac arrest, systemic oxygen delivery is insufficient^32,33^, and lactate levels increase owing to the hypoperfusion of vital organs. Therefore, the lactate level could be a marker of circulatory failure and a prognostic marker of cardiac arrest. Furthermore, even under aerobic conditions, lactate levels may be elevated by many factors, e.g., stress hyperlactatemia^34,35^, systemic inflammatory response^34,36^, myocardial stunning^10^, and mitochondrial dysfunction^10,37^. These multiple factors may be associated with the critical condition of OHCA because cardiac arrest is a highly heterogeneous entity. Whatever the cause, elevated lactate level might be a prognostic marker of poor outcomes in OHCA patients.

Serum lactate level before ROSC may be a candidate as a prognostic predictor of cardiac arrest. In the clinical settings of OHCA patients during CPR, physicians need to decide whether to initiate resuscitation strategies such as ECMO. Furthermore, the clinical decision to terminate resuscitation must be made during CPR. Serum lactate levels can be immediately and objectively measured even during CPR, enabling physicians to understand OHCA patients’ conditions even if their prehospitalization information is unknown.

Lactate levels before ROSC in our study were higher than those in previous studies regarding OHCA. Nonetheless, we showed that very high lactate levels during CPR were related to worse outcomes among OHCA patients in our study. Together with other variables, lactate levels during CPR may help clinicians’ clinical decision whether to proceed to the further resuscitation strategies in addition to the initial conventional CPR or to stop CPR during resuscitation efforts for OHCA patients.

Interestingly, our subgroup analysis demonstrated no relationship between lactate levels during CPR and survival among OHCA patients with initial shockable rhythm. On the other hand, in patients with non-shockable rhythm, the receiver operating characteristic of lactate level was 0.75, and the prognostic value was relatively high. The previous animal model showed some carotid blood flow was maintained during ventricular fibrillation^38^. Therefore, in patients with shockable rhythm, some blood flow might be maintained. And the tissue oxygen delivery might be relatively maintained even when the lactate level rises under aerobic conditions. Further research is required to clarify the mechanisms underlying the role of lactate levels. Thus, early prognostic stratification of OHCA patients should not be based on a single factor, and serum lactate level should be used as one of the predictors in conjunction with others in a multidisciplinary assessment.

## Limitations

This study has several inherent limitations. First, because this was an observational study, the protocols, such as the timing of blood work, were not strictly defined in each hospital. In addition, we could not determine the blood collection sites during CPR or whether arterial or venous blood was collected. However, there was no remarkable difference in lactate levels between collection sites or between blood samples from the artery and vein in a previous study^39^. Second, this registry did not obtain detailed information about each patient, their past medical history, medications, or comorbidities. For example, liver failure as comorbidity, medication, fluid infusion, and vasopressor use during CPR might affect lactate dynamics. If patients with high lactate levels had these conditions, the results would have tended toward poor outcomes. Third, blood parameters other than lactate levels were excluded from our final analyses because they were measured simultaneously with blood gas data. As serum lactate level would be influenced by other blood data, it would be beneficial to investigate the effect of combining other biomarkers and establish a prognostic prediction model of OHCA with multiple factors. Fourth, one of the important limitations of this study was that the interpretation of the lactate level by the medical staff in charge of the patient might have influenced the decision-making with regard to the resuscitation strategies: such an attitude could have intrinsically reduced the outcome of the patients with the higher lactate values, like a self-fulfilling prophecy. Therefore, this issue may bias our results to a significant extent. Finally, unmeasured confounding factors may have influenced the association between the lactate levels during CPR and OHCA outcomes.

## Conclusions

Using the large multicenter registry of OHCA, we demonstrated that high serum lactate levels during CPR were associated with poor 1-month survival, especially in patients with non-shockable rhythm, suggesting the role of serum lactate level as a prognostic marker of outcomes for OHCA patients before ROSC.

## Supplementary Information

Supplementary tables

## Data Availability

The dataset supporting the conclusions of this article is available from the corresponding author on reasonable request.

## Abbreviations

CAG: Coronary angiography
CCMC: Critical care medical center
CI: Confidence intervals
CPC: Cerebral Performance Category
CPR: Cardiopulmonary Resuscitation
CRITICAL: Comprehensive Registry of Intensive Care for OHCA Survival
ECMO: Extracorporeal membrane oxygenation
EMS: Emergency-medical-services
FDMA: Fire and Disaster Management Agency
OHCA: Out-of-hospital cardiac arrest
OR: Odds ratios
ROC: Receiver operating characteristics
ROSC: Return of spontaneous circulation
TTM: Target temperature management
