# Supplementary material for: Association between serum lactate level during cardiopulmonary resuscitation and survival in adult out-of-hospital cardiac arrest: a multicenter cohort study
Source: Sci Rep. 2021 Jan 15;11:1639. doi: 10.1038/s41598-020-80774-4 (PMC7810983; doi:10.1038/s41598-020-80774-4)
Supplement: Supplementary file 1 — Supplementary tables [file 41598_2020_80774_MOESM1_ESM.docx]

**Association Between Serum Lactate Level During Cardiopulmonary Resuscitation and Survival in Adult Out-of-Hospital Cardiac Arrest: A Multicenter Cohort Study**

Norihiro Nishioka, Daisuke Kobayashi, Junichi Izawa, Taro Irisawa, Tomoki Yamada, Kazuhisa Yoshiya, Changhwi Park, Tetsuro Nishimura, Takuya Ishibe, Yoshiki Yagi, Takeyuki Kiguchi, Masafumi Kishimoto, Toshiya Inoue, Yasuyuki Hayashi, Taku Sogabe, Takaya Morooka, Haruko Sakamoto, Keitaro Suzuki, Fumiko Nakamura, Tasuku Matsuyama, Yohei Okada, Satoshi Matsui, Atsushi Hirayama, Satoshi Yoshimura, Shunsuke Kimata, Takeshi Shimazu, Tetsuhisa Kitamura, Takashi Kawamura, Taku Iwami, on behalf of the CRITICAL Study Group Investigators

**Supplementary table 1.** Characteristics after OHCA patients by first documented rhythm

OHCA, out-of-hospital cardiac arrest; ROSC, return of spontaneous circulation; AED, automated external defibrillator; CPR, cardiopulmonary resuscitation; EMS, emergency medical services; IQR, Inter quartile range

*Comparisons between the two groups were evaluated with Kruskal-Wallis tests for continuous variables and X^2^ test for categorical variables.

**Supplementary Table 2.**  The results of sensitivity analysis

*Adjusted for age, sex, bystander witness, bystander CPR, first documented rhythm at the scene, prehospital advanced airway management, prehospital adrenaline administration, time from EMS call to lactate measurement, extracorporeal membrane oxygenation, coronary angiography, and target temperature management

ROSC, return of spontaneous circulation; OR, odds ratio; CI, confidence interval
